# A Note Concerning the Action of Chloroform

**Published:** 1890-12

**Authors:** Dudley Wilmot Buxton

**Affiliations:** Member of the Royal College of Physicians, Administrator of Anæsthetists in University College Hospital, the Hospital for Women, Soho Square, and the London Dental Hospital.


ARTICLE III.

### A NOTE CONCERNING THE ACTION OF CHLOROFORM.

BY DUDLEY WILMOT BUXTON, M. D., B. S. LOND.

**Member of the Royal College of Physicians, Administrator of Anæsthetists in University College Hospital, the Hospital for Women, Soho Square, and the London Dental Hospital.**

The interesting paper from the pen of Mr. Gaddes, which appeared in your issue of September 1st, cannot, I think be permitted to pass without comment. It is hardly necessary to point out that while every one feels a debt of gratitude has been incurred through the public spirit of Surgeon-Major Laurie, at whose suggestion his Highness the Nizam of Hyderabad entered upon the enlightened course of subsidising a Commission of Scientists to investigate a physiological problem of the first importance to the suffering human race, yet a large number of persons more or less skilled alike in the science and practice of Anæsthetics are unable to accept as proven the conclusions arrived at by the Commission. Not a little perplexity and misunderstanding will be spared if the history of the subject is more clearly known, and if it be made evident what really was the state of knowledge antecedent to the formation of the first Commission. Again, careful distinction should be made between the physiological work of the Commission, of its kind most excellent, and the theories built up from these experiments. The legend of the Silver

Shield bears with striking force upon the matter in hand, for while upon the one hand the Commission may regard the proving of experiments as establishing facts of one kind, viewed from the rigidly impartial view of physiological criticism, these same experiments may lend themselves in other minds to a very different class of facts.

Simpson, disliking the clinging odor of ether in confinements, determined, at the suggestion of Mr. Waldie, of Liverpool, to try the effect of the so-called "Chloric ether," which he did. In company with Drs. Keith and Duncan, Sir James, then Dr. Simpson, inhaled chloroform, and, profiting by the snoring of one colleague and the involuntary struggle of the other, he decided that chloroform was worthy of extended trial. It was introduced to the profession by him in his historic pamphlet, "Notice of a New Anæsthetic Agent as a Substitute for Sulphuric Ether in Surgery and Midwifery," November, 1847. However, more or less experiment was being made with chloroform in different parts of the world at this time. Flourens, in the March of the same year, had employed "chloric ether" as an anæsthetic for the lower animals, but came to the conclusion that it was too dangerous for general use.

Glover,* as early as 1842, employed chloroform, and found that absolute paralysis of the heart muscle in mammals followed its injection into the jugular vein. Sibson,† again clearly pointed out that in human beings death from chloroform occurred through heart failure. He wrote: "The heart, influenced by the poison (chloroform), ceased to contract, not from cessation of respiration, for the heart in asphyxia will beat from one to three minutes after respiration has ceased, but from immediate death of the heart." And later on the same author says: "We are obliged then, to conclude from the experience of these cases that in man the death is usually instantaneous, and

---

* Edinburgh Medical and Surgical Journal, Vol. 58.
† London Medical Gazette, Vol. xiii., 1848.

due, as every instantaneous death is, to paralysis of the heart. In animals [? the lower animals] the death is usually due to paralysis of the muscles of respiration," and that is of course the result of paralysis of the respiratory centres in the medulla. But the most thorough investigation of the action of chloroform in its early days is due to Dr. Snow, whose work was so accurate, so careful and thorough, that one finds most modern experimenters merely travelling in his footsteps, amplifying possibly, but not superseding, a physiological investigation worthy of the highest encomium. In 1885, a Commission was appointed in Paris, and it tendered its report to the Society of Emulation, and came to the conclusion (an important one when it be borne in mind that France was then under the influence of Flourens' adverse criticism) that chloroform, when administered to the lower animals, kills them by stopping respiration, and that the heart beats on after respiration has ceased. About this time Mr. Thomas Wakley performed similar experiments in this country and came to a like conclusion. Thus we see that all these investigations admit that among the lower animals the most usual mode in which death results is through failure of respiration, and that the heart stops subsequently to this. Snow, commencing his research at this stage of our knowledge, showed that what appeared to be a contradiction was in fact a perfectly reconcilable law. Chloroform kills, contends this observer, when its vapour strength in the respired air reaches a certain strength; given to men or the lower animals, if this point is not reached the vital processes remain unimpaired. At the present day this statement requires modification, for there seems to be strong evidence to show that prolonged chloroformization (that is, prolonged contact of chloroform with the tissues) may lead to fatty degeneration, and even bring about post-anæsthetic death from that cause.* But even here Snow

* Thiem & Fischer, "Ueber todliche Nachwirkung des Chloroforms, 1889.

seems to have been before us, for he says †: "If animals [? the lower animals] are kept for a very long time under the deep influence of chloroform, they become ultimately exhausted, the circulation and respiration are gradually weakened, and cease nearly together." The experimenters who came to the conclusion that chloroform could not kill directly through the heart had guarded against the use of higher strengths of vapour than formed Snow's safe percentage, and hence their conclusions were erroneous and amounted in fact only to this: When giving chloroform in such a way as to ensure a strength of less than 5 per cent. animals do not die from primary heart failure. And this is very much what we shall presently see is the contention of the Chloroform Commission of Hyderabad, nor will experts gainsay it, although they demur, and I think justly, when the Commissioners venture to generalize and say: Therefore chloroform never kills primarily through the heart muscle. Snow further showed that high percentages (8 or 10) will even in the lower animals produce primary syncope and death. He makes the following pertinent remark anent deaths of dogs, &c., occurring during experiments: "I have, indeed, been informed of several instances in which animals died in a sudden, and what was thought unaccountable, manner, whilst chloroform was given to prevent pain and struggles which would be occasioned by physiological experiments. In these cases there is no doubt the heart was paralysed, but the experimenters were often too intent on other matters to observe the circumstance." It is curious that the Hyderabad Commissioners state that while their animals never died from primary heart failure, yet, deaths did occur from some unexplained cause. "The fatal result was brought about either by neglecting to watch the condition of the respiration during or after the administration of chloroform, . . . . or from a reckless administration of chloroform, in the endeavor to check or prevent struggles. * These words would seem to lend themselves to two interpretations, firstly, that the "reck-

† On Anæsthetics, p. 109.

* "Lancet," p. 158, vol. i., 1890, "Accidental Death."

less administration," leads to the use of a high percentage of vapor, which determined primary heart failure; secondly, that the animals died in some cases, from reflex syncope, the result of shock, evinced by their struggles, a contention which would seem to discountenance the view of the Commission, that animals partly anæsthetized feel no shock, and so are not liable to reflex syncope. It is not proposed to go to any great length into the subject, so I must refrain from any attempt at exhaustive quotation from Snow's pregnant remarks, but will sum them in the following quotation.† Speaking of the mode of death in a large number of cases, the particulars of which he gives, he says: "In all the cases in which the symptoms which occurred at the time of death are reported there is every reason to conclude, as shown above, that death took place by cardiac syncope or arrest of the action of the heart. In forty of these cases the symptoms of danger appeared to arise entirely from cardiac syncope, and were not complicated by the overaction of the chloroform on the brain. It was only in four cases that the breathing appeared to be embarassed and arrested by the effect of chloroform on the brain and medulla oblongata, at the time when the action of the heart was arrested by it; and only in one of these cases that the breathing was distinctly arrested by the effect of the chloroform, a few seconds before that agent also arrested the action of the heart. * * * Chloroform has the effect of suddenly arresting the action of the heart when it is mixed with the respired air to the extent of 8 or 10 per cent. or upwards; and we must therefore conclude that in the fatal cases of its inhalation, the air the patients were breathing just before the accidents occurred contained this amount of vapour." Snow's teaching, then, amounted to this—chloroform kills through cardiac syncope, induced by a too concentrated vapour of chloroform, or by an excessive accumulation of chloroform in the blood, giving rise to paralysis of the vital centres in the Medulla Oblongato, and causing cessation of respiration first, and heart failure second, and this may occur even when small doses of chloroform are given when care is

† Ibid, p. 217.

not taken to ensure due elimination of the poison. It is obvious that the first cause of death may come into action at any point of time during the administration of chloroform while the second cause would operate only in the later stages of the anæsthetisation. In 1864 a Committee was appointed by the Royal Medico Chirurgical Society of London to investigate the Physiological action of chloroform, and a number of eminent surgeons and physicians took part in the work, being assisted by the skill and special knowledge of Mr. Clover.* Its conclusions are thus summarized: "Chloroform at first increases the force of the heart's action, this effect is slight and transient." "When complete anæsthesia is produced by chloroform, the heart in all cases acts with less than its natural force. . . . The strongest doses of chloroform vapour when admitted freely into the lungs, destroy animal life by arresting the action of the heart." "By moderate doses of chloroform the heart's action is much weakened for some time before death ensues, respiration generally, but not invariably, ceases before the action of the heart, and death is due both to the failure of the heart's action and to that of the respiratory function."

Yet another Commission undertook the investigation of the action of anæsthetics in 1880, this time appointed by the British Medical Association.† Mainly experimental in its operations, this Commission accumulated a number of interesting and highly important facts. The experiments were performed by skilled physiologists working in their laboratories with every appliance at hand, so that we may fairly consider the conclusions at which they arrived as worthy of the most careful consideration, if not of credence. The report says: "Chloroform may cause death in dogs, either by primarily paralysing the heart or the respiration." "In most cases, respiration stops before the heart's action." Further, there is the following important statement: "Without going into detail, we may say that it soon became apparent to us that chloro-

* See "Transac. Roy. Med. Chir. Soc." for that year.

† See the "Brit. Med. Journal," Dec. 15th, 1880, and June 14th, 1890.

form, administered to dogs and rabbits, has a disastrous effect upon the respiratory centres; it is easy to kill one of these animals by pushing the chloroform until respiration is paralysed. In observing the rate of the heart during these experiments, it could often be determined by auscultation, that its contractions were maintained after respiration had ceased. It was apparent, however, that even when failure of respiration was more directly the cause of death, the heart was, to some extent, simultaneously affected; and there were even cases in which the heart appeared to fail at least as soon, if not before, the breathing." Again, the report runs: "The chief dangers" [of chloroform] "are (i) sudden stoppage of the heart; (ii) reduction of the blood pressure; (iii) alteration of the pulse-respiration ratio and sudden cessation of the respiration. "We showed (see Report, 1880) that chloroform vapor has a paralysing effect upon the muscular tissue of the heart, and, indeed, upon all kinds of protoplasm, when directly applied."

While engaged upon the investigation of the action of chloroform, ether, alcohol, ethidene dichloride, bromide of ethyl, and methylene, upon protoplasm, and especially upon that forming the heart muscle of frogs, I perfused the detached heart with both normal saline and blood solution, in which were varying doses of chloroform, and watched the result. The fluid, of course, circulated freely through the heart, and that organ would beat uniformly for hours unless the chloroform, or what not, was introduced into the circulating medium. When, however, chloroform, even in minute doses, was poured into it, and so reached directly the heart muscle, its beat became less in force and soon ceased. If, however, fresh circulating fluid were then perfused so that the chloroform were washed away, no recovery took place, the heart muscle was permanently damaged. This was a marked contrast to the effect of ether.

The Glasgow Committee further examined the heart in situ by opening the thorax and maintaining artificial respiration, and found "where chloroform was given, there is a most serious effect upon the heart; the right ventricle almost immediately becomes distended, the heart presently stops, with the right ventricle engorged with blood." "Ether may be given for an indefinite period, without interference with the heart." Upon the important subject of blood pressure under chloroform, the Report says that "besides the general fall of blood pressure, (which, be it remarked, betokens an incipient vascular failure,) at certain stages of chlorof rm narcosis, there may be sudden falls in blood pressure, due to interference with the heart's action, and no one can deny that this is a serious state of matters."

The next chapter of our history brings us to the establishment of the first Hyderabad Chloroform Commission. The report of this was not fully published in the English professional journals, and hence some reluctance was expressed as to the acceptance of conclusions based upon premises only imperfectly made known. So to meet this objection the Second Hyderabad Chloroform Commission was, through the generosity of His Highness the Nizam of Hyderabad, established, and Dr. Lauder Brunton was asked by the editors of the *Lancet* to join it as a representative. This Commission repeated the work done by its predecessor, although upon wider lines. Still its report may be taken as representing the opinions and facts on which these opinions were based, alike of the First and Second Hyderabad Chloroform Commissions.

The main issues to which these Commissions addressed themselves may be stated as follows:—1. How does chloroform kill? (*a*) through respiration failure alone, as taught by Syme and Simpson; or (*b*) through primary heart failure; or (*c*) through either of these, according to circumstances. Further attention was directed to subsidiary matters, among which we may note (*a*) the effect of chloroform upon blood pressure; (*b*) effects of shock under chloroform.

The experiments were carefully made upon dogs, goats, monkeys, and other animals, and as a result conclusions were arrived at that these animals died in every case in which they were carefully watched, through failure of respiration, and in no instance was primary heart failure met with. Had the Commissioners stopped here, we should probably have had no occasion to gainsay their "finding," but they did not. They formulated a series of conclusions, applying their facts derived mainly from pariah dogs, not to pariah dogs in the aggregate, but to human beings, and those, by the way, living under the most diverse conditions. Much mischief has already been done by the indiscriminate publication in the professional and lay press, both here and in America, of these "conclusions," without any attempt either to reproduce or examine critically the experiments from which they are supposed to follow. There is a wide gap in the animal scale between pariah dogs and Europeans, and no one conversant with the behavior of the lower animals under anæsthetics and that of human beings under the same circumstances, would be content to admit that generalizations from one to the other were worthy of more than the most guarded admission.

Again, the conclusions of the Hyderabad Commission are at variance with similar observations made upon the lower animals in this country, and as the experimenters were at least of equal manipulative skill, and possessed of competent physiological knowledge, we are bound to say that the conclusions are of only limited application, and do not apply to less tropical countries. As I have showed above, by quotation from the report, the Commission lost several animals from "accidental deaths," which deaths we may believe were due to heart failure from overdosage.

Again, the evidence brought forward by the Hyderabad Commission concerning chloroform death amounts to this: (i) when death occurred and the phenomena accompanying it were observed, it was due to paralysis of respiration, and heart failure did not take place until some seconds after cessation of respiration; (ii) no death among the animals experimented upon was observed to occur from primary heart failure The animals numbered in all about 500, but many were experimented upon in other ways. Now this is quite in keeping with what other experimentalists have found. Deaths under chloroform are stated to occur about once in every 2,500 or 3,000 persons subjected to the anæsthetic, so that it would not have been any matter of surprise if the Commission had not lost a single animal of their 500 or so, but they did lose them. Again, of the deaths (1 in 3,000 say) a proportion are due to carelessness or ignorant anæsthetising, and some to shock

(this the Commission would deny, but, as I shall show later on, their contention falls short of proof), while some are undoubtedly cases in which respiration stops before or simultaneously with the cardiac syncope, so that when all these deductions are made, we shall find the number of instances of primary cardiac syncope per thousand represented by a small fraction. Such being the case—and these facts would certainly be vouched for by anæsthetists of wide experience, such as Snow, Clover, Richardson—we are compelled to regard even positive evidence arrived at by the examination of so few as 500 animals as of slight value, as against evidence furnished by hundreds of thousands of administrations to human beings; and when we come to negative evidence—that the Commission did not meet with a single case among the lower animals of primary cardiac syncope—we are still more strongly impelled to regard the conclusions based upon such evidence as unreliable and wholly unsatisfactory.

There is, however, yet another strong contention to be urged against the results at which the Hyderabad Commissioners arrived, and that is that other observers working in the same lines have arrived at results quite at variance with those obtained in India. It would be out of place here to attempt to explain such discrepancies, by variation in the stamina of the animals used,* the protection of an environing uniformly high temperature, &c., as it is rather the object of the present communication to compare results than to harmonize or explain their divarications.

It has been pointed out above that the Glasgow Commission undoubtedly met with primary heart failure, just as Snow had many years before, and Professor Wood † says, "Chloroform is capable of causing death either by primarily arresting the respiration or primarily stopping the heart. . . ."

The experiments of Prof. McKendrick and Prof. Wood were like those of Surgeon-Major Laurie and Dr. Lauder Brunton, made upon dogs, cats, &c., so that there is no reason to account for their results being so diametrically opposite.—*London Dental Record.*

(*To be concluded.*)

---

* Although it is at least interesting in this connection to note that no less an authority than Professor Wood, speaking at the International Congress in London, 1881, stated that he found a great difference obtained in American and English dogs as to their behaviour when experimented upon with drugs.

† International Medical Congress ln Berlin, 1890. Address of Anæsthetics, "British Med. Journal, No. 1,546, p. 386.

